# Cardiorespiratory fitness is associated with fMRI signal in right cerebellum lobule VIIa Crus I and II during spatial navigation in older adult women

**DOI:** 10.3389/fnagi.2022.979741

**Published:** 2022-11-23

**Authors:** Kathryn L. Kern, Stephanie A. McMains, Thomas W. Storer, Scott D. Moffat, Karin Schon

**Affiliations:** ^1^Department of Anatomy & Neurobiology, Boston University Aram V. Chobanian & Edward Avedisian School of Medicine, Boston, MA, United States; ^2^Center for Systems Neuroscience, Boston University, Boston, MA, United States; ^3^Center for Memory and Brain, Boston University, Boston, MA, United States; ^4^Cognitive Neuroimaging Center, Boston University, Boston, MA, United States; ^5^Men’s Health, Aging, and Metabolism Unit, Brigham and Women’s Hospital, Boston, MA, United States; ^6^School of Psychology, Georgia Institute of Technology, Atlanta, GA, United States; ^7^Department of Psychological and Brain Sciences, Boston University, Boston, MA, United States

**Keywords:** aging, cardiorespiratory fitness, cerebellum, fMRI, navigation

## Abstract

Spatial navigation is a cognitive skill critical for accomplishing daily goal-directed behavior in a complex environment; however, older adults exhibit marked decline in navigation performance with age. Neuroprotective interventions that enhance the functional integrity of navigation-linked brain regions, such as those in the medial temporal lobe memory system, may preserve spatial navigation performance in older adults. Importantly, a well-established body of literature suggests that cardiorespiratory fitness has measurable effects on neurobiological integrity in the medial temporal lobes, as well as in other brain areas implicated in spatial navigation, such as the precuneus and cerebellum. However, whether cardiorespiratory fitness modulates brain activity in these regions during navigation in older adults remains unknown. Thus, the primary objective of the current study was to examine cardiorespiratory fitness as a modulator of fMRI activity in navigation-linked brain regions in cognitively healthy older adults. To accomplish this objective, cognitively intact participants (*N* = 22, aged 60–80 years) underwent cardiorespiratory fitness testing to estimate maximal oxygen uptake ($\overset{\cdot }{\text{V}}$O_2max_) and underwent whole-brain high-resolution fMRI while performing a virtual reality navigation task. Our older adult sample demonstrated significant fMRI signal in the right and left retrosplenial cortex, right precuneus, right and left inferior parietal cortex, right and left cerebellum lobule VIIa Crus I and II, right fusiform gyrus, right parahippocampal cortex, right lingual gyrus, and right hippocampus during encoding of a virtual environment. Most importantly, in women but not men (*N* = 16), cardiorespiratory fitness was positively associated with fMRI activity in the right cerebellum lobule VIIa Crus I and II, but not other navigation-linked brain areas. These findings suggest that the influence of cardiorespiratory fitness on brain function extends beyond the hippocampus, as observed in other work, to the cerebellum lobule VIIa Crus I and II, a component of the cerebellum that has recently been linked to cognition and more specifically, spatial processing.

## Introduction

Successful spatial navigation is accomplished by a series of brain regions that together, dynamically encode, maintain, and update spatial information in order to support goal-directed behavior and accurately form navigational memories (Brown and Chrastil, 2019). It is well-established that this system undergoes age-related changes in older adults, a phenomenon that has been exhibited through studies of behavior (Harris and Wolbers, 2012; Zhong and Moffat, 2018; Merhav and Wolbers, 2019) and brain function (Meulenbroek et al., 2004; Moffat et al., 2006; Antonova et al., 2009). Critically, cardiorespiratory fitness modulates performance on spatial navigation tasks (Nauer et al., 2020b) and structural integrity of navigation-linked brain regions (Colcombe et al., 2003; Erickson et al., 2009; Zlatar et al., 2015; Williams et al., 2017) in older adults. Nonetheless, whether cardiorespiratory fitness modulates brain activity in these regions during spatial navigation task performance in older adults remains unknown.

Electrophysiology experiments and human neuroimaging studies have indicated that the complex process of spatial navigation is conducted by multiple brain regions. Early work from rodent electrophysiology studies proposed the entorhinal cortex—hippocampus circuit as a probable primary constituent of the neural machinery underlying a cognitive, or spatial, map of the current environment (O’Keefe and Dostrovsky, 1971). In additional experiments, this was further evidenced by spatially selective single cells, including place cells in the hippocampus (O’Keefe, 1979, 1976), grid cells in the entorhinal cortex (Hafting et al., 2005; McNaughton et al., 2006; Killian et al., 2012), and border cells in the hippocampus and parahippocampal cortex more broadly (Solstad et al., 2008; Lever et al., 2009; Boccara et al., 2010). Furthermore, studies in humans using intracranial EEG recordings have identified single cells that respond to specific spatial locations and views of landmarks in the hippocampus and parahippocampal gyrus, reminiscent of place-selective cells in rodents (Ekstrom et al., 2003; Jacobs et al., 2013; Miller et al., 2013), as well as cells with grid-like spatial firing patterns in the entorhinal cortex (Jacobs et al., 2013; Nadasdy et al., 2017). Importantly, human fMRI studies have corroborated these findings by consistently demonstrating a role for the hippocampus and parahippocampal gyrus in spatial navigation (Maguire et al., 1999; Chrastil, 2013). However, this neuroimaging work has further indicated that successful spatial navigation is not carried out by these regions alone.

Over two decades of human fMRI studies have highlighted that multiple brain regions, including the parahippocampal gyrus, retrosplenial cortex, parietal cortex, and cerebellum, function alongside the hippocampus to process spatial information. These brain regions are involved in multiple mechanisms to accomplish spatial encoding, such as egocentric spatial processing, which encodes spatial relationships about the environment relative to one’s own position in space, and allocentric spatial processing, which encodes spatial relationships of objects within the environment in relation to one another. The collective efforts of multiple human neuroimaging studies have elucidated how different brain regions are activated in association with various aspects of navigation. To this point, the hippocampus has largely been associated with spatially based navigation strategies, as significant hippocampal activation has been demonstrated during a viewpoint-independent spatial memory task (Parslow et al., 2004) and the use of a spatial strategy to navigate within in a virtual environment (Iaria et al., 2003; Bohbot et al., 2004). The parahippocampal cortex has been implicated in scene perception, as significant parahippocampal cortex activation has been demonstrated during encoding and recall of topographic information (Aguirre et al., 1996), passively viewing scenes (Epstein and Kanwisher, 1998), and navigating to a goal location based on landmark cues, rather than self-referential, spatial cues (Zhang and Ekstrom, 2013). Activation in the retrosplenial cortex has been associated with both exposure to allocentric spatial information and self-motion cues (Epstein and Higgins, 2007; Iaria et al., 2007; Auger and Maguire, 2013; Burte et al., 2018), and activation in the parietal cortices has been shown during the perception and integration of self-motion cues and landmarks, or environmental cues (Kravitz et al., 2011; Lester et al., 2017). Finally, the cerebellum is consistently coactivated with the hippocampus, medial parietal cortices, and medial prefrontal cortices during spatial navigation (Iglói et al., 2015) and has been associated with tracking rotational self-motion while monitoring one’s position and orientation in space (Chrastil et al., 2017). This body of literature suggests that the medial temporal lobe functions in concert with other regions including the retrosplenial cortex, parietal cortex, and cerebellum to collectively support spatial navigation.

Despite the importance of spatial navigation for daily functioning within a complex environment, older adults demonstrate age-related decline in the capacity for accurate encoding and retrieval of spatial memories. Behaviorally, previous work has shown that older compared to young adult humans travel longer distances to find the hidden platform in a virtual Morris Water Maze task (Moffat and Resnick, 2002), perform worse in distance reproduction, rotation reproduction, and triangle completion (i.e., path integration) tasks (Harris and Wolbers, 2012), and demonstrate age-related deficits in allocentric memory and in updating memories after egocentric encoding (Merhav and Wolbers, 2019). Functional neuroimaging studies indicate that older compared to young adults demonstrate decreased or absent activation in the hippocampus, parahippocampal gyrus, and retrosplenial cortex during various tasks requiring allocentric navigation (Meulenbroek et al., 2004; Moffat et al., 2006; Antonova et al., 2009), and that decreased activation in regions such as the parahippocampal gyrus and retrosplenial cortex was associated with poorer navigational accuracy (Moffat et al., 2006). In addition, another study indicated that older compared to young adults demonstrate decreased grid-cell-like representations in the entorhinal cortex, which was associated with behavioral impairment on intrinsic self-motion-related computations (Stangl et al., 2018). These differences in activation patterns observed in older compared to young adults may represent a shift away from hippocampally dependent computations and toward extra-hippocampal strategies (Colombo et al., 2017; Lester et al., 2017; Li and King, 2019), as aging has known effects on hippocampal integrity (Raz et al., 2005; Lister and Barnes, 2009). In support of this hypothesis, two fMRI studies indicated that older compared to young adults show a shift toward striatal-based response strategies during spatial navigation tasks, evidenced by activation in the caudate nucleus rather than the hippocampus (Konishi et al., 2013; Schuck et al., 2015). However, another study suggested that this shift toward striatal-based responses occurs in only a subset of older adults, as a proportion of their study sample demonstrated preserved use of spatial response strategies as evidenced by significant hippocampal activation during encoding (Konishi et al., 2013). Thus, it is possible that aging-associated alterations in activation patterns, specifically those indicating a shift away from hippocampally dependent strategies, may underlie the lower performance in spatial navigation tasks that are observed with aging. Neuroprotective interventions that preserve hippocampal integrity in aging, and thus, encourage continued reliance on allocentric navigation strategies, may ameliorate some of the previously reported age-related changes in spatial navigation.

One modifiable lifestyle factor that is a promising candidate for the modulation of neurobiological integrity in the medial temporal lobe memory system, as well as other brain areas implicated in spatial navigation, is cardiorespiratory fitness. In older adults, aerobic exercise training that increases cardiorespiratory fitness is associated with greater hippocampal volume (Erickson et al., 2011; Jonasson et al., 2017) and greater hippocampal blood flow (Burdette et al., 2010; Maass et al., 2015). In addition, cross-sectional work suggests that fitness is positively associated with tissue density and cortical thickness in brain regions that demonstrate age-related decline, such as the frontal, parietal, and temporal cortices (Colcombe et al., 2003; Williams et al., 2017). Cardiorespiratory fitness has also been positively associated with gray matter density of the left cerebellum in older adults (Zlatar et al., 2015). Collectively, these studies provide compelling evidence for a relationship between cardiorespiratory fitness and the structural and functional integrity of brain regions responsible for successful navigation.

Importantly, several studies have also examined the relationships between fitness, spatial navigation task performance, and brain activation during virtual navigation. Greater cardiorespiratory fitness is associated with better performance on a virtual Morris Water Maze task in male adolescents (Herting and Nagel, 2013) and with mitigated age-related performance reductions in a spatial route disambiguation task in an adult lifespan sample (Nauer et al., 2020b). In middle-aged men and women, increased cardiorespiratory fitness through a 6-month exercise training program is associated with greater activation in widespread brain regions including the hippocampus, parahippocampal gyrus, retrosplenial cortex, and precuneus, among others, during successful spatial learning (Holzschneider et al., 2012). However, to our knowledge, whether cardiorespiratory fitness modulates brain activity during navigation of a virtual environment in cognitively healthy older adults remains unknown.

Therefore, the objective of the current study was to examine the relationships between cardiorespiratory fitness and blood oxygen level dependent (BOLD) signal in navigation-linked brain regions in cognitively healthy older adults. We hypothesized that cardiorespiratory fitness modulates BOLD signal in navigation-linked brain regions, specifically within the frontal, parietal, and temporal cortices, given that the previously published literature suggests that cardiorespiratory fitness enhances structural integrity in these regions. In order to test this hypothesis, we examined data from 22 cognitively healthy older adults who underwent cardiorespiratory fitness testing and whole-brain high-resolution fMRI while performing a virtual reality allocentric navigation task (Moffat et al., 2006) established in the cognitive aging literature.

## Materials and methods

### Participants

All participant data for the current study were acquired from an NIH-funded study from our laboratory (ClinicalTrials.gov Identifier: NCT02775760) (Kern et al., 2020; Rosario et al., 2021). We recruited older adults aged 60–80 years who were non-smoking, fluent in English, and sedentary (defined as participating in less than 30 min 3 days per week of physical activity that is moderate or high intensity) (Pescatello and American College of Sports Medicine, 2014). Importantly, exclusion criteria included history of conditions that affect cognitive function or may be associated with permanent brain damage, such as neurological disorders, psychiatric disorders, or severe stress; and past or present conditions that are contra-indicators for participation in cardiorespiratory fitness testing or training, such as heart, circulatory, or respiratory conditions, musculoskeletal conditions (without primary care physician clearance), or electrolyte disorders. Other exclusion criteria included poor vision that could not be corrected; presence of an acute infection; diagnosis of metabolic conditions, cancer, and/or severe anemia; use of cardioactive or psychoactive drugs; self-reported drug abuse or alcohol misuse; and/or contra-indicators for MRI participation, including claustrophobia, non-removable ferro-magnetic metal in or on the body, and susceptibility to extreme motion sickness (which may be provoked by virtual navigation tasks performed in the MRI scanner). Our resulting sample consisted of 22 healthy older adult participants (16 women).

### Overview of experimental design

**FIGURE 1:**
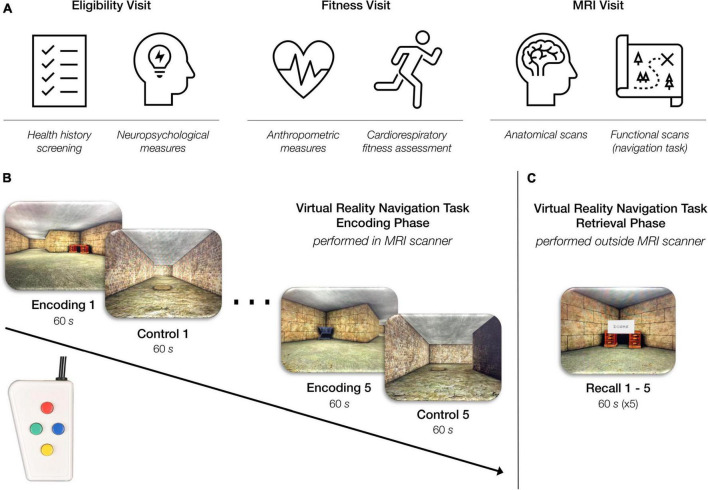
Overview of study procedures. **(A)** Participants came in for three study visits: an eligibility visit, a fitness visit, and an MRI visit. **(B)** *Virtual reality navigation task: encoding phase (inside the MRI scanner*). During the “encoding” condition of the task, participants were instructed to navigate through a series of rooms and interconnected hallways with the objective of learning the location of six objects placed within the environment. During the “control” condition of the task, participants were instructed to move through a hallway as quickly as possible while also traveling over a series of floor markers. The task alternated between the “encoding” and “control” condition every 60 s for a total of 10 min. Participants completed this phase of the task using a four-button, diamond-configuration response device to move in the virtual space. **(C)** *Virtual reality navigation task: retrieval phase (outside the scanner*). Participants began at one object in the virtual environment and were instructed to navigate to another object using the shortest possible route. Upon correctly locating the specified object, the participant was instructed to find another object. This continued throughout the duration of the 60-s recall block. Each participant completed five 60-s recall blocks consecutively.

All study procedures and protocols were compliant with the Code of Ethics set forth by the World Medical Association and were approved by the Institutional Review Board at the Boston University (BU) Medical Campus. Participants completed three study visits at BU Charles River Campus (Figure 1A). The Eligibility Visit (approximately 2.5 h in duration) took place at the BU Cognitive Neuroimaging Laboratory and included informed consent, health history screening, and neuropsychological assessments. The Fitness Visit (approximately 2.5 h in duration) took place at the BU Fitness and Recreation Center and included anthropometric measurements and a cardiorespiratory fitness assessment. The MRI Visit (approximately 4 h in duration) took place at the BU Cognitive Neuroimaging Center and included structural and functional MRI and cognitive testing. Chronologically, the Eligibility Visit always came first given its importance for examining inclusion and exclusion criteria. After the Eligibility Visit was completed and the participant was determined eligible, the scheduling of the Fitness Visit and MRI Visit was based on participant and facility availability. However, due to the known acute effects of exercise on cognition (Tomporowski, 2003; Suwabe et al., 2017), all participants completed the MRI Visit either before or 24 h after the Fitness Visit.

### Neuropsychological assessments

All participants completed standard neuropsychological assessments at the Eligibility Visit. In order to assess executive function, processing speed, and attention, we evaluated participants’ performance on the Victoria Stroop Test (VST) (Strauss et al., 2006) and the Trail Making Test (TMT) Version A and TMT B, respectively (Strauss et al., 2006). In order to assess subjective spatial cognition, participants completed the Santa Barbara Sense of Direction Scale (SBSOD) (Hegarty, 2002). Finally, in order to screen participants for evidence of cognitive impairment or dementia, we evaluated participant performance on the Dementia Rating Scale-2 (DRS-2) (Mattis, 1976). These measures were included to characterize our study sample as cognitively normal using population normative data (Au et al., 2004; Pedraza et al., 2010; Hankee et al., 2016).

### Cardiorespiratory fitness assessment

In exercise physiology, the gold-standard measure of cardiorespiratory fitness is maximal oxygen uptake ($\overset{\cdot }{\text{V}}$O_2max_, mL/kg/min) (Mitchell et al., 1958; Wagner, 1996). In the current study, as in our laboratory’s previous work (Kern et al., 2020; Nauer et al., 2020a,b; Rosario et al., 2021), we obtained an estimate of this measure. Participants performed a submaximal incremental treadmill test [terminated at 85% of age-predicted maximal heart rate (Pescatello and American College of Sports Medicine, 2014)] in accordance with a modified Balke protocol (Hagberg, 1994; Cooper and Storer, 2001; Pescatello and American College of Sports Medicine, 2014). We selected this approach to help ensure the safety of our older adult participants due to the non-clinical setting of the assessments. Furthermore, to ensure the data collected were not biased by participant fatigue or the effects of caffeine on heart rate, we instructed all participants not to partake in strenuous physical activity for 24 h before fitness testing, nor to consume caffeine the 3 h before fitness testing.

The submaximal incremental treadmill test protocol utilized in our laboratory includes three separate stages: 3 min of warm-up, 8–12 min of data collection, and 3 min of cool-down. During the data collection period of the test, each participant walked on the treadmill at their pre-determined, fastest comfortable walking pace while a study staff member incrementally increased the treadmill grade at the end of each 1-min effort. In order to ensure participant safety, two study staff members were present throughout the duration of the test in order to (1) continually monitor the participant’s heart rate, (2) measure the participant’s blood pressure after every 3 min of effort (Pescatello and American College of Sports Medicine, 2014), and (3) ask the participant their perceived level of effort using the 6–20 Borg Rating of Perceived Exertion scale (Borg, 1982) after every 3 min of effort. In addition, a study staff member recorded the participant’s heart rate within the last 5 s of every 1-min effort as the primary outcome measure of this test. This was accomplished using a heart rate watch (Polar, model A300) that wirelessly paired to a heart rate monitor worn using a chest strap by the participant (Polar, model H1).

After test completion, the participant’s oxygen uptake for each 1-min treadmill effort was evaluated using a metabolic calculation for the estimation of energy expenditure during walking (Equation 1) (Pescatello and American College of Sports Medicine, 2014):


(1) $$\overset{\cdot }{\text{V}}{\text{O}}_{2}=(0.1\;\text{mL/kg/min}\;\text{.}\;S)\;+\;(1.8\;\text{mL/kg/min}\;\text{.}\;S\;.\;G)\;+\;3.5\;\text{mL/kg/min}$$

In this equation, $\overset{\cdot }{\text{V}}$O_2_ is oxygen uptake (mL/kg/min), *S* is speed (m/min), *G* is percent grade expressed as a decimal, and 3.5 mL/kg/min corresponds to resting energy expenditure (Pescatello and American College of Sports Medicine, 2014). We used this equation to calculate the $\overset{\cdot }{\text{V}}$O_2_ demonstrated by the participant in each minute of effort during data collection. We then, exploited the known linear relationship between heart rate and $\overset{\cdot }{\text{V}}$O_2_ (Wasserman, 2012) to estimate each participant’s $\overset{\cdot }{\text{V}}$O_2max_ using their age-predicted maximum heart rate. Using this methodology, we estimated $\overset{\cdot }{\text{V}}$O_2max_ for all participants, which was thus, used to operationalize cardiorespiratory fitness in the present study.

Lastly, in order to appreciate the fitness of the participants within the context of national reference standards stratified by age and sex, we interpolated all estimates of $\overset{\cdot }{\text{V}}$O_2max_ into age- and sex-specific fitness percentiles, as characterized by the Reference Standards for Cardiorespiratory Fitness Measured with Cardiopulmonary Exercise Testing (Kaminsky et al., 2015). These interpolated percentiles were included to consider the overall fitness of our sample in the context of national normative values but were not used for further analyses.

### MRI procedures

#### MRI data acquisition

Participants were scanned using a 3 Tesla Siemens MAGNETOM Prisma MRI scanner (Siemens Healthcare, Erlangen, Germany) equipped with a stock 64-channel head coil at BU Cognitive Neuroimaging Center. We used the Siemens Auto-Align tool to establish reproducible placement of the image fields of view. We collected two anatomical scans for each participant: a whole-brain structural T1-weighted magnetization-prepared rapid gradient multi-echo sequence [multi-echo MPRAGE (van der Kouwe et al., 2008); slices = 176 sagittal; TR (ms) = 2,200; TEs (ms) = 1.67, 3.5, 5.33, and 7.16; TI (ms) = 1,100; flip angle (degrees) = 7; voxel size (mm^3^) = 1.00 × 1.00 × 1.00; FOV read (mm) = 230; GRAPPA acceleration = 4], and a structural T2-weighted volume with high in-plane resolution [slices = 30; slice thickness (mm) = 2; phase encoding direction = R >> L; TR (ms) = 8,020; TE (ms) = 80; voxel size (mm^3^) = 0.4 × 0.4 × 2.0; FOV read (mm) = 150]. Additionally, for each participant, we collected 302 BOLD volumes during 10 min 4 s of the virtual reality navigation task encoding phase (described below). We acquired the functional images using a high-resolution T2*-sensitive echo planar imaging (EPI) pulse sequence that employed multiband RF pulses and Simultaneous Multi-Slice (SMS) acquisition (Feinberg et al., 2010; Moeller et al., 2010; Setsompop et al., 2012; Xu et al., 2013) [slices = 90; phase encoding direction = R >> L; TR (ms) = 2,000.0; TE (ms) = 30.0; spacing between slices (mm) = 0; SMS factor = 3; iPAT = 2 (Griswold et al., 2002); voxel resolution (mm^3^) = 1.50 × 1.50 × 1.50; FOV read (mm) = 162; flip angle (degrees) = 45].

#### Virtual reality allocentric navigation task

Our laboratory developed an adapted version of the virtual reality allocentric navigation task used in Moffat et al. (2006). Our allocentric navigation task (Figures 1B,C) was designed for use with a standard fMRI block design and included a navigation condition and a control condition which alternated every 60 s. One run of the task included five navigation blocks and five control blocks for a total of 600 s (10 min). In the navigation, or “encoding” condition, participants were presented with the same maze-like environment that included multiple rooms and interconnecting hallways. The maze-like environment contained six objects, and the participant was instructed to learn the location of the objects as well as how the hallways interconnected. In the control condition, participants were presented with a hallway-like environment and were instructed to move through the hallway as quickly as possible while also traveling over a series of floor markers. The task alternated automatically between the “encoding” and control conditions until the participant completed five blocks of each condition in the single run. Throughout the task, the participant navigated through the environment from the first-person perspective using a diamond-shaped button box (Current Designs Inc.; Philadelphia, PA), as shown in Figures 1B,C.

After completion of the encoding phase of the task in the MRI scanner, the participant completed the retrieval phase of the task outside the MRI scanner. In the retrieval portion of the task, we tested the participant’s memory by asking them to navigate to a specific object in the maze using the shortest possible route from a specific location. Upon correctly locating the specified object, the participant was instructed to reach a specific new object. This continued, and the participant was instructed to try to reach as many of the specified objects as is possible within the 60-s recall block. The retrieval phase of the task included five 60-s recall blocks. The behavioral results associated with the retrieval phase of the task are not examined in the current study.

#### Preprocessing and single-subject level analyses

We completed all preprocessing of anatomical and functional images using MRIcroGL (version 2017-07-14)^1^ and Analysis of Functional Neuroimages (AFNI) software (version 19.1.00) (Cox, 1996). First, all scans were converted from DICOM to NIfTI format using dcm2niix (Li et al., 2016) with MRIcroGL. Next, we performed preprocessing in AFNI which included slice time correction (*3dTshift*) and motion correction (*3dvolreg*) for the functional volumes, linear alignment of the anatomical to the functional dataset (*align_epi_anat.py*), and non-linear normalization of the anatomical dataset to an age-specific MRI brain template built from older adults aged 65–69 years (Fillmore et al., 2015; Richards et al., 2016) (*@auto_tlrc*). These interpolation steps were combined into a single catenated transformation in order to reduce interpolation (Polimeni et al., 2018). Notably, any timepoint with > 0.3 mm of motion was removed through censoring. Finally, the functional data were spatially smoothed with a 3 mm FWHM filter (*3dmerge*) and converted to percent signal change (*3dTstat*).

To perform single-subject level analyses, we used a canonical hemodynamic response function to model each condition (i.e., encoding or control) using one parameter (fixed shape) block stimuli of 59 s duration (*3dDeconvolve*) where the control condition served as the baseline. We also included an “instruction” regressor of 1 s duration to account for the initial second of each block that cued participants to block type. This command generated single-subject coefficients for the “encoding” vs. “control” condition that were used in group analyses.

Finally, we performed quality assurance on our data to identify any participants with a high level of motion. We used four motion metrics, as recommended by AFNI’s software (Cox, 1996) to evaluate image quality and motion levels. These metrics included average censored motion (average motion magnitude across non-censored TRs), maximum censored displacement (maximum displacement among non-censored volume pairs), censor fraction (fraction of total TRs censored due to the > 0.3 mm threshold), and average temporal signal-to-noise ratio (TSNR). Any participant who was identified as a statistically significant outlier in two or more of the four motion metrics was excluded from group-level analyses.

### Statistics and group-level analyses

To characterize the demographics, neuropsychological performance, and physiology of our study sample, we performed statistical analyses using RStudio (Version 1.2.5001).^2^ First, we examined all variables (*demographic*—age, education; *neuropsychological measures*—VST ratio, TMT B/A ratio, SBSOD score, DRS-2 total raw score, DRS-2 memory raw score; *physiology*—estimated $\overset{\cdot }{\text{V}}$O_2max_, BMI, resting heart rate, estimated $\overset{\cdot }{\text{V}}$O_2max_ percentile) for normality using Shapiro Wilk tests of normality. Next, we summarized all variables that were normally distributed using mean and standard deviation (SD) and summarized all variables that were not normally distributed using median and interquartile range (IQR). In addition, given that we were interested in sex-specific relationships between cardiorespiratory fitness and brain function outcome measures, we also summarized all variables separately for women and men. Due to the small number of men whose data were included in the current study (*N* = 6), we did not test for sex differences within our study sample. However, we did examine effect size of sex differences using Hedges g for small samples. Finally, we examined the relationship between estimated $\overset{\cdot }{\text{V}}$O_2max_ and all neuropsychological measures using simple Pearson’s correlations and Spearman’s rank correlations based on normality. These correlations were performed both across the whole sample (including both men and women) and in subgroups separated by sex. These data summaries were included to further characterize our study sample by their demographics, performance on neuropsychological tests, and physiology.

Next, to characterize the pattern of activation associated with the performance of a virtual reality allocentric navigation task in our older adult sample, we ran standard univariate analyses with a *t*-test using the *3dttest*++ command. In order to correct for the large number of multiple comparisons, we used AFNI’s non-parametric “Equitable Thresholding and Clustering” (ETAC) (Cox, 2019). Using ETAC, we performed an additional 3 mm of Gaussian blurring, which is additive in the square of FWHM blurring, such that the final spatial smoothing of the data was 4.24 mm. In addition, we included the non-blurring parameters of nearest neighbor NN = 2, 2-sided *t*-tests, H powers hpow = 2, and a *p*-threshold of 0.001. We selected this *p*-threshold to optimize spatial specificity and thus, increase the likelihood that clusters were localized to specific anatomical regions, rather than crossing defined anatomical boundaries (Woo et al., 2014). This group-level analysis resulted in an output dataset with mean beta values for each regressor specified in the original single-subject level analyses paired with an associated Z-score. To move the group-level output dataset from the space of the age-specific MRI brain template (Fillmore et al., 2015; Richards et al., 2016) into the MNI space, we first, registered the age-specific MRI brain template to MNI space using non-linear warping (*auto_warp.py*), and then, applied the associated non-linear transformation matrix to the group-level output dataset (*3dNwarpApply*). Finally, we identified the coordinates of the voxel with the maximum intensity value of each cluster using the *3dClusterize* function in AFNI. We used the Desikan-Killiany atlas (Desikan et al., 2006) for significant clusters in the cerebral cortex and Eickhoff-Zilles maximum probability maps on MNI-152 from post-mortem analysis (Eickhoff et al., 2006) for significant clusters in the cerebellum.

To test the prediction associated with the primary objective of our study (i.e., cardiorespiratory fitness is associated with BOLD signal in navigation-linked brain regions), we utilized two separate approaches. Our first approach was to perform a second *t*-test using the *3dttest*++ command while including estimated $\overset{\cdot }{\text{V}}$O_2max_ as a statistical covariate, as in other work (Holzschneider et al., 2012). This allowed us to examine the relationship between cardiorespiratory fitness and navigation-linked brain activation (betas for encoding vs. the control condition) throughout the brain. In order to control for false positives globally, we used *3dClustSim* (Cox et al., 2017), a more conservative non-parametric method that preceded ETAC and works with covariates. Group-level clusters were considered significant if they met the clustering threshold identified by this program for bi-sided thresholding at the level of *p* = 0.010, alpha = 0.05, nearest neighbor NN = 2 (faces or edges touch). For our second approach, we used the *3dmaskave* command in AFNI to extract the average timeseries from the voxels in each previously identified significant cluster (i.e., clusters that demonstrated significantly different activation patterns in the encoding and control conditions of the task). After extracting these beta coefficients, we next pulled the data into RStudio to run linear regression analyses to examine the relationships between estimated $\overset{\cdot }{\text{V}}$O_2max_ and the navigation-linked brain region beta coefficients, and included age and sex as covariates. In addition, given the established sex-differences in the previously published literature examining relationships between cardiorespiratory fitness, brain structural and functional integrity (Varma et al., 2015, 2016; Dimech et al., 2019; Kern et al., 2020), and cognition (Colcombe and Kramer, 2003; Barha et al., 2017), we ran linear regression analyses to examine the relationships between estimated $\overset{\cdot }{\text{V}}$O_2max_ and the navigation-linked brain region beta coefficients in women only (*N* = 16) including age as a covariate. Due to the small number of men whose data were included in the current study (*N* = 6), we did not examine interactions between estimated $\overset{\cdot }{\text{V}}$O_2max_ and sex in predicting navigation-linked brain region beta coefficients. For the same reason, we did not examine the relationship between estimated $\overset{\cdot }{\text{V}}$O_2max_ and navigation-linked brain region beta coefficients in men only. Importantly, we corrected all *p*-values for multiple comparisons using Benjamini & Hochberg (false discovery rate) (FDR) corrections (Benjamini and Hochberg, 1995) as applied by the *p.adjust* function in R.

## Results

### Participant demographics, neuropsychological performance, and physiology

We collected full datasets, including demographic, cardiorespiratory fitness, and neuroimaging data, from 31 older adult participants. Four participants were excluded because they did not reach 85% of their maximum heart rate during cardiorespiratory fitness testing, thus, failing to complete the assessment. One participant was excluded due to an incidental finding on the structural MRI scan in one of our regions of interest. One participant failed single-subject analysis at the *3dDeconvolve* step due to data censoring (97% of data censored; insufficient data for estimating regression parameters). Another four participants were removed from analyses given that they were statistical outliers in two or more of the four motion metrics collected during the quality assurance step of the single-subject level analyses. After removing these nine participants, our resulting sample consisted of 22 healthy older adult participants (16 women). The demographics, neuropsychological measures, and physiological data for all participants are reported in Table 1. Importantly, two of the 16 women indicated that they were taking estrogen and/or hormone replacement therapy, and one of the 16 women indicated that she was taking letrozole post-mastectomy.

**TABLE 1:** Demographic, neuropsychological, and physiological measures for a sample of 22 older adults (aged 60–80 years).

	All participants (*N* = 22)	Women (*N* = 16)	Men (*N* = 6)	Hedges g (*N* = 22)
**Demographics—*mean* ± *SD***				
Age (years)	67.68 ± 5.56	67.00 ± 5.27	69.50 ± 6.41	
**Demographics—*median (IQR*)**				
Education (years)	17.5 (4)	17 (2.25)	20 (0.75)	
**Neuropsychological measures—*mean* ± *SD***				
Victoria Stroop test ratio	1.97 ± 0.38	1.90 ± 0.34	2.14 ± 0.44	−0.612
Trail making test B/A Ratio	2.31 ± 0.78	2.26 ± 0.79	2.43 ± 0.79	−0.208
SBSOD score	65.86 ± 18.15	67.38 ± 16.09	61.83 ± 24.10	0.289
**Neuropsychological measures—*median (IQR*)**				
DRS-2 total raw score	142 (2)	142 (2)	141.5 (1.75)	−0.197
DRS-2 memory raw score	24.5 (1)	24.5 (1.25)	24.5 (1)	−0.149
**Physiology—*mean* ± *SD***				
Estimated $\overset{\cdot }{\text{V}}$O_2max_ (mL/kg/min)	30.74 ± 6.67	28.95 ± 6.50	35.53 ± 4.72	−1.038
BMI (kg/m^2^)	27.88 ± 5.75	27.59 ± 6.32	28.65 ± 4.23	−0.174
**Physiology—*median (IQR*)**				
Resting heart rate (beats per minute)	71 (13.5)	67 (15.25)	74 (3.75)	−0.305
Estimated $\overset{\cdot }{\text{V}}$O_2max_ percentile (range 1–99)	97 (18.5)	98 (10)	83.5 (10.5)	0.446

BMI, body mass index; DRS-2, Dementia Rating Scale-2; SBSOD, Santa Barbara Sense of Direction; $\overset{\cdot }{\text{V}}$O_2max_, maximal oxygen uptake. The Hedges g reported here reflects the effect size of sex differences for the reported measures.

#### Demographics

First, we examined age and education using Shapiro Wilk tests of normality. Age was normally distributed, whereas education was not normally distributed (Supplementary Table 1). Variables are summarized in Table 1. Regarding age, the mean ± SD age (years) of our study sample was 67.68 ± 5.56 years. Regarding education, the median (IQR) years of education of our study sample was 17.5 (IQR = 4). Notably, our participants reported being very highly educated, with 90.9% of the study sample reporting that their highest degree attained was a bachelor’s degree or higher (20 out of 22 participants; 16–20+ years of education) and 63.6% of the study sample reporting that their highest degree attained was a master’s degree, professional degree (MD, JD, DDS, etc.), or doctorate (14 participants; 17–20+ years of education).

#### Neuropsychological measures

Next, we examined the neuropsychological measures using Shapiro Wilk tests of normality. The VST ratio, TMT B/A ratio, and SBSOD score were normally distributed, whereas DRS-2 total raw score and DRS-2 memory raw score were not normally distributed (Supplementary Table 1). Variables are summarized in Table 1. Regarding the VST, the mean ± SD ratio of our study sample was 1.97 ± 0.38; and for the TMT, the mean ± SD B/A ratio of our study sample was 2.31 ± 0.78. For the SBSOD, the mean ± SD score of our study sample was 65.86 ± 18.15. Finally, regarding the DRS-2 scores: for the DRS-2 total raw score, the median (IQR) score of our study sample was 142 (2), and for the DRS-2 memory raw score, the median (IQR) score of our study sample was 24.5 (1). The measures of center for all neuropsychological measures were within the cognitively normal range for healthy older adults, suggesting that enrolled participants were cognitively unimpaired. Furthermore, for TMT A, TMT B, and VST ratio, all participants individually scored within 1.5 SD of the age group mean as determined by adult normative values (Au et al., 2004). In addition, as specified by the eligibility criteria for the DRS-2, there were no participants whose Age-Corrected MOANS Scaled Score (AMSS) or Age- and Education-Corrected MOANS Scaled Score (AEMSS) was 8 or below (indicative of “Mild Impairment”). Table 1 shows the breakdown of neuropsychological measures by sex, as well as the associated effect sizes to estimate the magnitude of the observed sex differences.

#### Physiology

**FIGURE 2:**
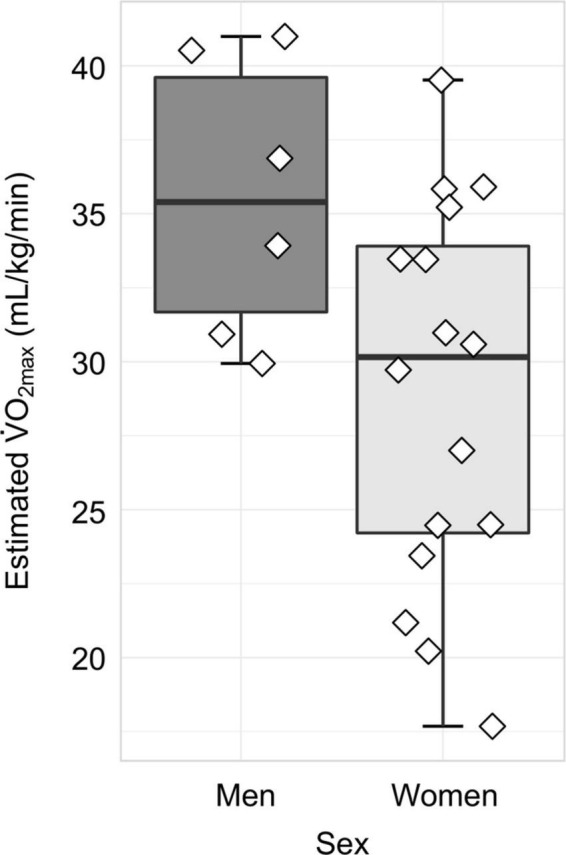
Distribution of cardiorespiratory fitness, as measured by estimated $\overset{\cdot }{\text{V}}$O_2max_, separated by sex. In women (*N* = 16), the mean ± SD estimated $\overset{\cdot }{\text{V}}$O_2max_ was 28.95 ± 6.50 mL/kg/min, whereas in men (*N* = 6), the mean ± SD estimated $\overset{\cdot }{\text{V}}$O_2max_ was 35.53 ± 4.72 mL/kg/min.

Finally, we examined all physiological variables using Shapiro Wilk tests of normality. $\overset{\cdot }{\text{V}}$O_2max_ and BMI were normally distributed, whereas resting heart rate and estimated $\overset{\cdot }{\text{V}}$O_2max_ percentile were not normally distributed (Supplementary Table 1). Variables are summarized in Table 1. Regarding estimated $\overset{\cdot }{\text{V}}$O_2max_, the mean ± SD was 30.74 ± 6.67 mL/kg/min. The distribution of estimated $\overset{\cdot }{\text{V}}$O_2max_ values separated by sex is displayed in Figure 2. For BMI, the mean ± SD was 27.88 ± 5.75; for resting heat rate, the median (IQR) beats per minute was 71 (13.5); and for estimated $\overset{\cdot }{\text{V}}$O_2max_ percentile, the mean (IQR) percentile was 97 (18.5). For the breakdown of physiological measures by sex, as well as the associated effect size estimates, please see Table 1.

Collectively, the measures of center for estimated $\overset{\cdot }{\text{V}}$O_2max_ and estimated $\overset{\cdot }{\text{V}}$O_2max_ percentile demonstrate that our sample is relatively high fit compared to national normative values (Kaminsky et al., 2015).

#### Correlation between neuropsychological measures and estimated $\overset{\cdot }{\text{V}}$O_2max_

After summarizing the neuropsychological and physiological variables, we next, sought to clarify whether any significant correlations existed among the neuropsychological measures and estimated $\overset{\cdot }{\text{V}}$O_2max_. Using simple Pearson correlations for normally distributed variables, no significant associations were observed between estimated $\overset{\cdot }{\text{V}}$O_2max_ and VST ratio, TMT B/A ratio, or SBSOD score across the whole sample (Supplementary Table 2). Using Spearman’s correlations for non-normally distributed variables, no significant associations were observed between estimated $\overset{\cdot }{\text{V}}$O_2max_ and DRS-2 total raw score or DRS-2 memory raw score across the whole sample (Supplementary Table 2). Furthermore, these correlations remained non-significant when examined in subgroups separated by sex (Supplementary Table 2).

### Encoding task condition is associated with robust brain activity in navigation-linked brain regions

**FIGURE 3:**
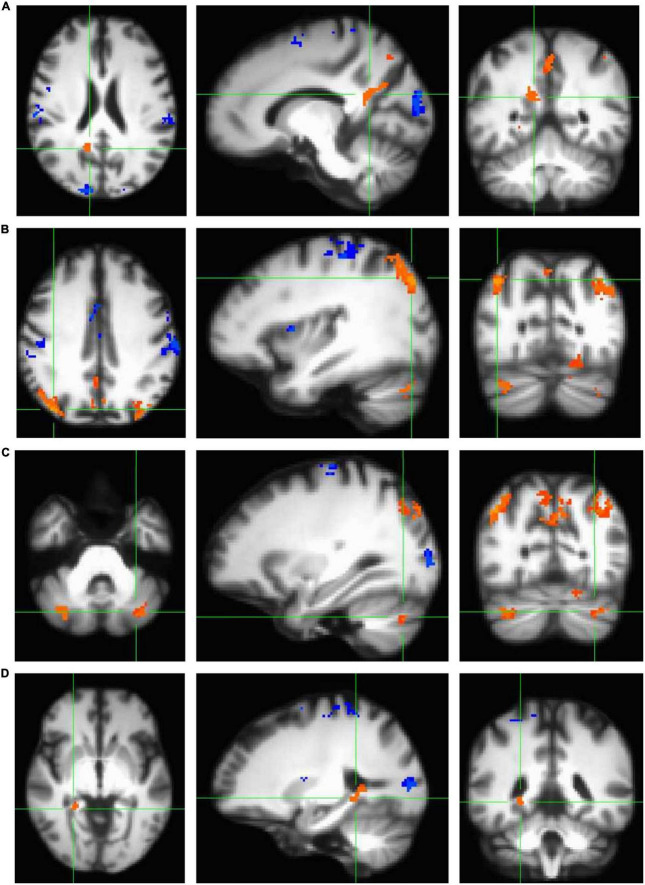
Clusters that demonstrated statistically significant activation (*p* < 0.001) during the navigation task in encoding > control conditions (orange) and control > encoding conditions (blue) as measured using a standard univariate analysis in AFNI (*3dttest*++ with ETAC). All clusters shown here survived correction for multiple comparisons and are demonstrated on the age-specific MRI brain template warped to MNI space. Note that all four panels **(A–D)** show a significant encoding > control cluster in three planes, listed left to right: axial (left = right hemisphere), sagittal (left = anterior), and coronal (left = right hemisphere). **(A)** Significant cluster comprising the bilateral retrosplenial cortex and right precuneus. **(B)** Two significant clusters comprising bilateral inferior parietal cortex. **(C)** Two significant clusters comprising the bilateral cerebellum (lobule VIIa Crus I and II). **(D)** Significant cluster comprising the medial temporal areas, including right fusiform gyrus, right parahippocampal gyrus (and more specifically, parahippocampal cortex), right lingual gyrus, and right hippocampus.

In order to characterize the pattern of activation associated with the performance of a virtual reality allocentric navigation task in our older adult sample, we examined whole-brain, high-resolution BOLD signal during an established spatial navigation task. Our sample of older adults demonstrated robust brain activity in widespread brain regions commonly associated with navigation (Table 2 and Figure 3). Clusters that demonstrated significantly greater activation in the encoding condition compared to the control condition (encoding > control) at the *p* < 0.001 level included the right and left isthmus of the cingulate cortex (retrosplenial cortex), right precuneus, right and left inferior parietal cortex, right and left cerebellum in lobule VIIa Crus I and II, right fusiform gyrus, right parahippocampal gyrus (and more specifically, the parahippocampal cortex), right lingual gyrus, and right hippocampus (Table 2 and Figure 3). Clusters that demonstrated significantly greater activation in the control condition compared to the encoding condition (control > encoding) at the *p* < 0.001 level are reported in Supplementary Table 3. All of the clusters reported in Table 2 and Supplementary Table 3 survived multiple comparison correction and were at least 100 voxels in size. These findings demonstrate that the encoding condition of the task, which required active navigation of a virtual environment with the goal of learning the location of specific objects, activated multiple brain regions that play a key role in spatial representation, including the bilateral retrosplenial cortex, bilateral inferior parietal cortex, bilateral cerebellum, right parahippocampal cortex, and right hippocampus.

**TABLE 2:** Clusters that demonstrated significantly greater brain activation (*p* < 0.001) in the encoding condition compared to the control condition (encoding > control) that are 100 voxels or greater in size.

Cluster		Maximum intensity (MI)			Region of interest		
		
Number	Size (Voxels)	X	Y	Z	Distance from MI (mm)	Hemisphere	Regions
1	1,213	5.1	−47.4	11.3	0	R	Isthmus of the cingulate cortex (Retrosplenial cortex)
					5	L	Isthmus of the cingulate cortex (retrosplenial cortex)
					5	R	Precuneus
2	846	45.6	−71.4	27.8	0	R	Inferior parietal cortex
3	581	−42.9	−77.4	27.8	0	L	Inferior parietal cortex
4	247	−11.4	−81.9	−29.2	0	L	Lobule VIIa Crus I (hemisphere)
					3	L	Lobule VIIa Crus II (hemisphere)
5	155	32.1	−71.4	−41.2	0	R	Lobule VIIa Crus I (hemisphere)
					1	R	Lobule VIIa Crus II (hemisphere)
6	155	−33.9	−66.9	−41.2	0	L	Lobule VIIa Crus II (hemisphere)
					2	L	Lobule VIIa Crus I (hemisphere)
7	133	26.1	−42.9	−8.2	2	R	Fusiform gyrus
					3	R	Parahippocampal gyrus
					3	R	Lingual gyrus
					4	R	Hippocampus

All clusters survived correction for multiple comparisons. Maximum intensity (MI) coordinates are reported in MNI space. X, left-right direction; Y, posterior-anterior direction; Z, inferior-superior direction.

### Across the whole sample of men and women, there was no relationship between estimated $\overset{\cdot }{\text{V}}$O_2max_ and BOLD signal in navigation-linked brain regions

To test the prediction associated with the primary objective of our study (cardiorespiratory fitness modulates BOLD signal in navigation-linked brain regions, specifically within the frontal, parietal, and temporal cortices, given that the previously published literature suggests that cardiorespiratory fitness enhances structural integrity in these regions), we first, performed a group-level *t*-test within the whole sample of men and women (*N* = 22) to examine differences between the encoding and control conditions of the task while including estimated $\overset{\cdot }{\text{V}}$O_2max_ as a statistical covariate, as in other work (Holzschneider et al., 2012). This allowed us to examine the relationship between cardiorespiratory fitness and navigation-linked brain activation. No clusters survived correction for multiple comparisons [cluster size threshold *k* ≥ 131; bi-sided thresholding, *p* = 0.010, alpha = 0.05, nearest neighbor NN = 2 (faces or edges touch); largest cluster size *k* = 67 across the whole sample]. In order to examine if this may be due to the influence of age and sex on these relationships, we performed an additional group-level *t*-test with estimated $\overset{\cdot }{\text{V}}$O_2max_, age, and sex as covariates, as in other work (Hayes et al., 2017). However, again, no clusters survived correction for multiple comparisons [cluster size threshold *k* ≥ 431; bi-sided thresholding, *p* = 0.010, alpha = 0.05, nearest neighbor NN = 2 (faces or edges touch); largest cluster size *k* = 59].

Given that this first approach to correcting for multiple comparisons across the whole brain was selected to optimize sensitivity (true positive rate) and control Type I error, we next, performed a second approach using beta coefficient extraction to optimize specificity (true negative rate) and control Type II error (Lindquist and Mejia, 2015). For this approach, we extracted the beta coefficient average for the clusters that demonstrated significantly greater activation in the encoding condition compared to the control condition (Table 2 and Figure 3). At this point, we performed linear regression analyses to examine if cardiorespiratory fitness predicted ROI beta coefficient while controlling for age and sex. In summary, across the whole sample of men and women (*N* = 22), none of the relationships between estimated $\overset{\cdot }{\text{V}}$O_2max_ and ROI beta coefficients while controlling for age and sex were significant. Across the whole sample, there was no relationship between estimated $\overset{\cdot }{\text{V}}$O_2max_ and beta coefficient in the right and left isthmus of the cingulate cortex (retrosplenial cortex) and right precuneus, the right inferior parietal cortex, the left inferior parietal cortex, the left cerebellum lobule VIIa Crus I and II (clusters 4 and 6), the right cerebellum lobule VIIa Crus I and II, nor the right fusiform gyrus, parahippocampal cortex, lingual gyrus, and hippocampus (Supplementary Table 4).

### In women only, estimated $\overset{\cdot }{\text{V}}$O_2max_ is associated with BOLD signal in the right cerebellum lobule VIIa Crus I and II during navigation

**FIGURE 4:**
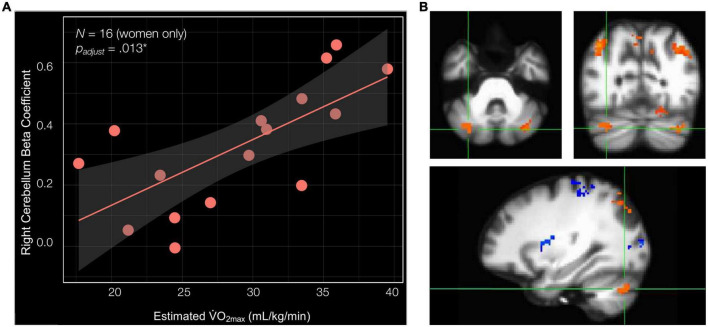
Cardiorespiratory fitness is associated with right cerebellum lobule VIIa Crus I and II beta coefficient in women. **(A)** Estimated $\overset{\cdot }{\text{V}}$O_2max_ is significantly associated with the beta coefficient of the cluster in the right cerebellum lobule VIIa Crus I and II (estimated $\overset{\cdot }{\text{V}}$O_2max_—β = 0.023, SE = 0.006, *t*[13] = 3.881, *p* = 0.002**, *p*_*adjust*_ for ROIs (7) = 0.013*, delta-R^2^ = 0.532). **(B)** Significant cluster comprised of the right cerebellum lobule VIIa Crus I and II shown in three planes, listed in order of top left, top right, and bottom: axial (left = right hemisphere), coronal (left = right hemisphere), and sagittal (left = anterior).

Previous publications have demonstrated relationships between cardiorespiratory fitness and hippocampal subfield structural integrity that are unique to women (Varma et al., 2015, 2016; Kern et al., 2020), as well as relationships between aerobic exercise and cognitive function that are unique to women (Colcombe and Kramer, 2003). Thus, we next ran linear regression analyses to examine the relationships between estimated $\overset{\cdot }{\text{V}}$O_2max_ and the navigation-linked brain region beta coefficients in women only (*N* = 16) including age as a covariate. Importantly, in women, estimated $\overset{\cdot }{\text{V}}$O_2max_ significantly positively predicted the beta coefficient of the cluster in the right cerebellum lobule VIIa Crus I and II [estimated $\overset{\cdot }{\text{V}}$O_2max_—β = 0.023, SE = 0.006, *t*[13] = 3.881, *p* = 0.002^**^, *p*_*adjust*_ for ROIs (7) = 0.013*, delta-R^2^ = 0.532] (Figure 4 and Supplementary Table 5). Estimated $\overset{\cdot }{\text{V}}$O_2max_ did not significantly predict ROI beta coefficient in any other cluster in women. These non-significant clusters included the right and left isthmus of the cingulate cortex and right precuneus, the right inferior parietal cortex, the left inferior parietal cortex, the left cerebellum lobule VIIa Crus I and II (clusters 4 and 6), and the cluster composed of the right fusiform gyrus, parahippocampal cortex, lingual gyrus, and hippocampus (Supplementary Table 5). Considered altogether, these findings suggest that estimated $\overset{\cdot }{\text{V}}$O_2max_ uniquely predicts brain activation in the right cerebellum lobule VIIa Crus I and II during allocentric navigation task performance in women.

## Discussion

The overall objective of the current study was to test the hypothesis that cardiorespiratory fitness modulates BOLD signal in navigation-linked brain regions, specifically within the frontal, parietal, and temporal cortices, in cognitively healthy older adults. During encoding of the virtual environment, our sample of older adults demonstrated significant BOLD signal in the right and left isthmus of the cingulate cortex (retrosplenial cortex), the right parahippocampal gyrus (and more specifically, the parahippocampal cortex), and the right hippocampus, which are brain regions that have been shown to exhibit either reduced or absent BOLD signal in cognitively healthy older adults during navigation (Meulenbroek et al., 2004; Moffat et al., 2006; Antonova et al., 2009), but may be active when older adults use a spatially based, rather than response-based, strategy during encoding (Konishi et al., 2013). In addition, our older adult sample demonstrated significant activation in the right precuneus, right and left inferior parietal cortex, and right and left cerebellum lobule VIIa Crus I and II. Most importantly, we present evidence for a positive relationship between cardiorespiratory fitness and BOLD signal in the right cerebellum lobule VIIa Crus I and II during encoding of the virtual environment in women. These data suggest that the influence of cardiorespiratory fitness-related neuroplasticity may extend beyond the hippocampus to the cerebellum lobule VIIa Crus I and II, which is known to play a role in sequence-based navigation.

### The plasticity of the cerebellum and its role in spatial processing

The cerebellum is not frequently the focus of human neuroimaging studies examining the influence of cardiorespiratory fitness on the neural correlates of cognition; however, there is a significant body of literature including animal and human studies that provide evidence for cerebellar plasticity in association with aerobic exercise or cardiorespiratory fitness. In rodents, exposure to physical exercise through wheel or treadmill running has been associated with angiogenesis in the paramedian lobule of the cerebellum (also known as lobule HVIIB in humans) (Black et al., 1990; Isaacs et al., 1992) and amelioration of age-related Purkinje cell loss across the cerebellum (Larsen et al., 2000). In older adults, one structural MRI study demonstrated that greater cardiorespiratory fitness was associated with greater gray matter density in the left cerebellum (Zlatar et al., 2015). Furthermore, a functional MRI study demonstrated that greater cardiorespiratory fitness was associated with greater BOLD signal in the cerebellum, among other regions, during an associative encoding task (Hayes et al., 2017).

Critically, this body of literature demonstrating a relationship between fitness and cerebellar plasticity is complemented by over a decade of rodent and human research that suggests a role for the cerebellum in spatial processing. In rodents, electrophysiology studies have demonstrated that cerebellar plasticity results in both modified hippocampal place cell activation and behavioral impairments when relying on self-motion cues to complete a navigation task (Rochefort et al., 2011). Moreover, more recent work has demonstrated that the hippocampus and anatomically connected regions of the cerebellum demonstrate synchronization of neural oscillations, providing a mechanism by which the cerebellum may influence hippocampal activation during functions such as spatial navigation (Watson et al., 2019). In humans, multiple fMRI studies have identified specific cerebellum lobules that are involved in spatial processing. Two seminal task-based fMRI studies demonstrated a role for cerebellum lobule VIIa Crus I and II in mental rotation tasks in young adult men (Stoodley et al., 2010, 2012). In addition, another task-based fMRI study demonstrated that lateralized recruitment of cerebellum lobule VIIa Crus I may be dependent upon the strategy employed for the navigation task, such that sequence-based navigation recruits the right cerebellum lobule VIIa Crus I, whereas place-based navigation recruits the left cerebellum lobule VIIa Crus I (Iglói et al., 2015). Collectively, these studies demonstrating a role for the cerebellum in spatial processing illuminate how a relationship between cardiorespiratory fitness and cerebellar plasticity may modulate spatial cognition.

Multiple neurobiological and systems-level mechanisms may underly the current study’s observed positive relationship between cardiorespiratory fitness and brain activity in the right cerebellum lobule VIIa Crus I and II during encoding of a virtual environment in women. As aforementioned, rodent work has demonstrated that both wheel and treadmill running is associated with amelioration of age-related Purkinje cell loss (Larsen et al., 2000) and angiogenesis (Black et al., 1990; Isaacs et al., 1992) in the cerebellum. It is possible that similar mechanisms occur in healthy older adults which thus, could affect cerebellar BOLD signal. In addition to these cellular mechanisms, functional neuroimaging studies have suggested that the bilateral cerebellum lobules VIIa Crus I and II may be functionally linked to the hippocampus, a brain region that demonstrates significant plasticity in association with aerobic exercise and cardiorespiratory fitness. One study in particular provided evidence to suggest that the right cerebellum lobule VIIa Crus I exhibits functional connectivity with the medial prefrontal cortex and left hippocampus, creating a non-motor loop that supports sequence-based navigation (Iglói et al., 2015). In accompaniment, these authors suggested that the left cerebellum lobule VIIa Crus I exhibits a functional link to the medial prefrontal cortex and right hippocampus, creating a non-motor loop that supports place-based navigation (Iglói et al., 2015). These proposed functional circuits are consistent with the previously published literature that demonstrates lateralized effects of cardiorespiratory fitness and/or aerobic exercise to the left hippocampus (Rosano et al., 2017; Wagner et al., 2017; Firth et al., 2018; Nauer et al., 2020a). Thus, our observed relationship between cardiorespiratory fitness and BOLD signal in the right cerebellum lobule VIIa Crus I and II may be modulated by the effect of cardiorespiratory fitness on the left hippocampus. Future studies should consider examining whether cardiorespiratory fitness modulates functional connectivity between the left hippocampus and right cerebellum lobule VIIa Crus I and II during virtual navigation in older adults to examine this mechanistic possibility.

### The association between cardiorespiratory fitness and right cerebellum lobule VIIa crus I and II BOLD signal was observed in women only

Finally, it is important to note that the current study’s observed relationship between cardiorespiratory fitness and BOLD signal in the right cerebellum lobule VIIa Crus I and II was found in women, not men. Because we had a low number of men enrolled in the study (*N* = 6), we did not statistically test for differences between the sexes.

Other studies in older adults have also demonstrated sex-dependent relationships between physical activity, cardiorespiratory fitness, and brain structure and function (Colcombe and Kramer, 2003; Varma et al., 2015, 2016; Kern et al., 2020). It is possible that the observed sex differences in the current study may be mediated by sex steroid hormones, neurotrophin levels, and the interactions between these molecules. A developing body of literature has indicated a role for sex steroid hormones, such as estradiol and testosterone, in the modulation of neuroplasticity mechanisms in the brain (Barha et al., 2017; Galea et al., 2017). Through neuroplasticity mechanisms and others, sex steroid hormones offer neuroprotection for multiple regions of the brain including the hippocampus and cerebellum (Siddiqui et al., 2016). One hypothesis that has been proposed (Brown et al., 2013) to explain why women compared to men may demonstrate a more pronounced benefit of exercise on brain integrity is in relation to the significant decrease in estrogen levels after menopause. This significant decrease in estrogen may provide an intervention point for postmenopausal women for exercise-related increases in sex hormones, such as testosterone (Janssen, 2016), to rescue the menopause-associated decrease in neuroprotection (Brown et al., 2013).

In addition to studies in humans, animal models have elucidated the molecular and cellular level interactions between sex steroid hormones, neurotrophins, and neuroplasticity modulation. One study demonstrated that whereas male compared to female mice exhibit significantly greater levels of BDNF in the frontal cortex, amygdala, and ventral hippocampus, female compared to male mice exhibit significantly greater levels of BDNF in the cerebellum (Zhu et al., 2006). And importantly, another study demonstrated that female compared to male rats exhibit a greater BDNF response to enriched environments that included a running wheel (Bakos et al., 2009). Altogether, these studies provide evidence for sex-moderated neurobiological mechanisms underlying observed sex-dependent relationships between cardiorespiratory fitness and brain plasticity in the current study and others. Future human neuroimaging work should consider examining levels of sex hormones and BDNF as possible mediators of sex-dependent relationships.

It is important to note that our findings exist alongside a sizeable body of literature demonstrating sex differences in maze learning strategies that are related to hormone levels (reviewed in Hegarty et al., 2022). In female rodents, studies have demonstrated that a low level of estrogen is associated with response-based learning strategies, whereas a high level of estrogen or estrogen replacement is associated with place-based strategies (Korol et al., 2004; Hussain et al., 2014). Similar results have been found in young adult humans, such that on a task designed to probe place vs. response strategies (Marchette et al., 2011), men were more likely to rely on place-based strategies, whereas women were more likely to rely on response-based strategies (Boone et al., 2018). Critically, it is likely that aging and more specifically, menopause, may influence the relationships between hormone levels and the neural circuitry underlying spatial navigation (Jacobs et al., 2016; Taylor et al., 2019). This further emphasizes the importance of examining sex hormones as a modulator of the observed sex-dependent relationships between cardiorespiratory fitness and brain plasticity in the current study and others.

### Our cognitively healthy older adult sample demonstrated significant brain activation in multiple navigation-associated regions

In addition to examining the relationship between cardiorespiratory fitness and BOLD signal during a spatial navigation paradigm, the current study also reports the brain activation patterns observed during the established spatial navigation task (Moffat et al., 2006) in our sample of cognitively healthy older adults. Notably, past studies examining activation patterns in navigation-linked brain regions have demonstrated robust activation in frontal regions, as well as decreased or absent BOLD signal in the hippocampus, parahippocampal gyrus, and retrosplenial cortex in older compared to young adults (Meulenbroek et al., 2004; Moffat et al., 2006; Antonova et al., 2009). Comparatively, our older adult sample exhibited significant activation in the right and left isthmus of the cingulate cortex (retrosplenial cortex), the right parahippocampal cortex, and the right hippocampus, all of which are regions that have been shown to exhibit reduced or absent BOLD signal in older adults compared to young adults during allocentric encoding paradigms (Meulenbroek et al., 2004; Moffat et al., 2006; Antonova et al., 2009). The data presented here do not include a young adult control group, and as such, it is still possible that our older adult sample would show lower BOLD signal compared to young adults.

Another possible explanation for the preserved BOLD signal in these regions is that the age-related shift away from hippocampally dependent navigation strategies occurs in only a subset of older adults, as demonstrated elsewhere (Konishi et al., 2013). A qualitative comparison of our results to that of other studies comparing navigation-related BOLD signal in young and older adults (Moffat et al., 2006) suggests that the activation pattern demonstrated by our older adult sample may more closely mirror that of young adults rather than that of older adults. It is possible that the activation patterns observed in our older adult sample may represent the preservation of more youthful neural function, which may be due to the stringent inclusion and exclusion criteria that were required for inclusion in the current study. Importantly, many of our eligibility criteria (e.g., smoking and freedom from diagnoses of arthritis, diabetes, and depression) are linked to correlates of “successful aging” (Martin et al., 2015), considered both in relation to maintenance of physical (Depp and Jeste, 2006; Jeste et al., 2010) and cognitive (Nyberg et al., 2012; Nyberg and Pudas, 2019) function. Future work should consider whether older adults demonstrating these correlates of successful aging are more likely to employ hippocampally dependent allocentric strategies than striatally dependent response-based strategies to solve spatial navigation tasks, thus, providing clarification as to why some populations of older adults are likely to demonstrate preserved hippocampal activation in aging.

### Limitations and considerations

There are several limitations of the current study that warrant acknowledgments. First, it is important to note that our older adult study sample demonstrated a selection bias toward very highly fit and highly educated older adults. The likelihood of our laboratory’s fitness and exercise studies to recruit highly fit older adults has been noted and discussed in our laboratory’s published work (Kern et al., 2020; Nauer et al., 2020b). This may in part be attributable to the stringent nature of our inclusion and exclusion criteria, which ensure participant safety during cardiorespiratory fitness testing, but are also likely to result in the exclusion of lower fit individuals. Examples of these inclusion and exclusion criteria include but are not limited to circulatory, respiratory, or musculoskeletal conditions, diabetes mellitus, and/or the use of medications for circulatory conditions, such as hypertension or hypercholesterolemia, that affect heart rate. In addition to highly fit participants, the current study demonstrated a selection bias toward very highly educated older adults. A high level of education is associated with the preservation of age-sensitive cognitive functions, such as episodic memory (Nyberg et al., 2012; Nyberg and Pudas, 2019), and furthermore, is associated with lower incidence of cardiovascular disease (Hoeymans et al., 1996; Liu et al., 2019). Thus, it is possible that the high education level of our sample resulted in the preservation of youthful neural function in our older adults. Future work should seek to widen the range of fitness levels and education levels of older adults included in their experiments to confirm that any observed fitness-related relationships are generalizable to the broader aged population.

In addition, it is also important to acknowledge that the current study is limited by the small sample size, the small number of men in the study sample, and the cross-sectional nature of the study. It is possible that the smaller study sample precluded us from observing significant activation in additional brain regions, for example, the left hippocampus. Moreover, the recruitment of additional men would have been critical to statistically examine sex differences in the observed fitness-related relationships. Groups in the future seeking to do similar fitness-related interventions should use recruitment strategies that will yield study samples that are balanced by sex. It is also noteworthy that the applications of the results of the current study are limited by the cross-sectional design. Longitudinal studies including an exercise training program can control for interindividual differences in cardiorespiratory fitness performance and aptitude, which is important due to the significant effect of genetics on fitness (Barbato et al., 1998; Teran-Garcia et al., 2008).

It is also critical to acknowledge that the two primary outcome measures of the current study, cardiorespiratory fitness as measured by estimated $\overset{\cdot }{\text{V}}$O_2max_ and fMRI BOLD signal, are both dependent upon the cardiovascular system. This limitation has been discussed in detail in our laboratory’s other published work (Kronman et al., 2020). In summary, this phenomenon is attributable to the fact that fMRI BOLD signal is a proxy measure of underlying neural activity that is observed through local changes in the cerebral metabolic rate of oxygen consumption, cerebral blood flow, and cerebral blood volume (D’Esposito et al., 2003). Thus, it is possible that cardiorespiratory fitness may influence these factors directly, rather than the neural activity that this proxy measure seeks to represent. It is noteworthy that our data indicated a unique relationship between cardiorespiratory fitness and BOLD signal in the right cerebellum lobule VIIa Crus I and II. This suggests that cardiorespiratory fitness affects either the underlying neural activity or the cardiovascular response profile of this specific brain region, rather than exerting widespread changes on the cardiovascular response profile of the whole cerebellum or whole brain. One way to control for this issue is the use of an internal control, i.e., measuring the change in BOLD signal in a longitudinal study design (D’Esposito et al., 2003). Future studies should consider these experimental design elements for examining the relationships between cardiorespiratory fitness and brain structure and function in older adults.

Finally, it is important to note that the primary objective of this study was to examine the relationship between cardiorespiratory fitness and BOLD signal in navigation-linked brain regions, not behavioral performance on a navigation task. As such, we are not able to draw conclusions regarding what the observed relationship means for navigation performance. We took this approach given that precise cognitive outcome measures are required to indicate whether fitness is associated with changes in cognition (Voss et al., 2019), and navigation is a complex cognitive skill carried out by multiple brain regions that perform heterogeneous functions (Brown and Chrastil, 2019). Thus, we instead sought to use fMRI as a tool to develop an insightful, data-based hypothesis regarding which specific component(s) of spatial cognition may be modulated by fitness. Our results presenting evidence for a positive relationship between cardiorespiratory fitness and BOLD signal in the right cerebellum lobule VIIa Crus I and II during encoding of the virtual environment in women provides a data-based hypothesis suggesting that fitness may influence sequence-based navigation (Iglói et al., 2015). Future work examining the relationship between cardiorespiratory fitness and navigation performance in aging should consider using precise, hypothesis-driven cognitive outcome measures developed to tax sequence-based navigation in order to examine whether fitness is associated with changes in cognition.

## Conclusion

The current study is among the first to examine how cardiorespiratory fitness modulates whole-brain BOLD signal during spatial navigation in cognitively healthy older adults. We provide a novel contribution to the existing literature by demonstrating that cardiorespiratory fitness is positively associated with BOLD signal in the right cerebellum lobule VIIa Crus I and II, a component of the cerebellum that is involved in cognitive function and more specifically, spatial processing, during virtual navigation in older adult women. This work indicates that cardiorespiratory fitness-related plasticity in the adult human brain extends beyond the structure and function of the hippocampal memory system. In addition, it begs the question of how exercise-related modifications in cerebellar BOLD signal may impact spatial navigation, a cognitive function that demonstrates marked decline in aging.

## Data availability statement

The raw data supporting the conclusions of this article will be made available by the authors, upon reasonable request.

## Ethics statement

The studies involving human participants were reviewed and approved by the Institutional Review Board at the Boston University Medical Campus. The participants provided their written informed consent to participate in this study.

## Author contributions

TS, SM, and KS designed the research study. KK performed the data collection, statistical analyses, and wrote the original draft of the manuscript. KK and SAM wrote preprocessing scripts. KS oversaw the project. All authors contributed to the final version of the manuscript and approved the submitted version.

## Footnotes

1
https://www.nitrc.org/projects/mricrogl

2
https://rstudio.com/
