# Supplementary material for: Cardiorespiratory fitness is associated with fMRI signal in right cerebellum lobule VIIa Crus I and II during spatial navigation in older adult women
Source: Front Aging Neurosci. 2022 Nov 23;14:979741. doi: 10.3389/fnagi.2022.979741 (PMC9727394; doi:10.3389/fnagi.2022.979741)
Supplement: Supplementary file 1 [file Data_Sheet_1.docx]

Supplementary Material

# Supplementary Figures and Tables

**Supplemental Table 1: Examining demographic, neuropsychological, and physiological variables for normality using Shapiro-Wilk tests of normality.** Abbreviations: BMI, body mass index; DRS-2, Dementia Rating Scale-2; SBSOD, Santa Barbara Sense of Direction; V̇O_2max_, maximal oxygen uptake. ^†^Denotes *p* < .100. *Denotes significance at the level of *p* < .050. **Denotes significance at the level of *p* < .010. ***Denotes significance at the level of *p* < .001.

|  | **All Participants**  **(*N* = 22)** | **Women**  **(*N* = 16)** | **Men**  **(*N* = 6)** |
| --- | --- | --- | --- |
| **Demographics –** |  |  |  |
| **Age (years)** | W = 0.951, *p* = .336 | W = 0.924, *p* = .195 | W = 0.965, *p* = .859 |
| **Education (years)** | **W = 0.871, *p* = .008**** | W = 0.913, *p* = .129 | **W = 0.634, *p* = .001**** |
| **Neuropsychological Measures –** |  |  |  |
| **Victoria Stroop Test Ratio** | W = 0.972, *p* = .746 | W = 0.985, *p* = .989 | W = 0.905, *p* = .407 |
| **Trail Making Test B/A Ratio** | W = 0.963, *p* = .544 | W = 0.978, *p* = .942 | W = 0.840, *p* = .131 |
| **SBSOD Score** | W = 0.944, *p* = .237 | W = 0.928, *p* = .231 | W = 0.952, *p* = .758 |
| **DRS-2 Total Raw Score** | **W = 0.898, *p* = .028*** | W = 0.888, *p* = .052^†^ | W = 0.958, *p* = .804 |
| **DRS-2 Memory Raw Score** | **W = 0.790, *p* < .001***** | **W = 0.796, *p* = .002**** | W = 0.822, *p* = .091^†^ |
| **Physiology –** |  |  |  |
| **Estimated V̇O_2max_ (mL/kg/min)** | W = 0.962, *p* = .540 | W = 0.96, *p* = .654 | W = 0.907, *p* = .418 |
| **BMI (kg/m^2^)** | W = 0.953, *p* = .363 | W = 0.931, *p* = .252 | W = 0.953, *p* = .768 |
| **Resting heart rate (beats per minute)** | W = 0.916, *p* = .063^†^ | **W = 0.852, *p* = .014*** | W = 0.876, *p* = .251 |
| **Estimated V̇O_2max_ percentile (range 1-99)** | **W = 0.763, *p* < .001***** | **W = 0.649, *p* < .001***** | W = 0.936, *p* = .630 |

**Supplemental Table 2: Examining correlations between neuropsychological measures and estimated V̇O_2max_.** Abbreviations: DRS-2, Dementia Rating Scale-2; SBSOD, Santa Barbara Sense of Direction; V̇O_2max_, maximal oxygen uptake. ^†^Denotes *p* < .100. *Denotes significance at the level of *p* < .050. **Denotes significance at the level of *p* < .010. ***Denotes significance at the level of *p* < .001.

|  | **Pearson's r; *p*-value** | | |
| --- | --- | --- | --- |
| **Neuropsychological Measures –** | **All Participants (*N* = 22)** | **Women (*N* = 16)** | **Men (*N* = 6)** |
| **Victoria Stroop Test Ratio** | *r* = .087; *p* = .701 | *r* = .144; *p* =.594 | *r* = -.678; *p* =.139 |
| **Trail Making Test B/A Ratio** | *r* = .240; *p* = .281 | *r* = .307; *p* =.248 | *r* = -.131; *p* =.805 |
| **SBSOD Score** | *r* = -.018; *p* = .937 | *r* = -.080; *p* =.768 | *r* = .420; *p* =.407 |
|  | **Spearman’s *ρ*; *p*-value** | | |
| **Neuropsychological Measures –** | **All Participants (*N* = 22)** | **Women (*N* = 16)** | **Men (*N* = 6)** |
| **DRS-2 Total Raw Score** | *ρ* = .058; *p* = .797 | *ρ* = -.003; *p* = .991 | *ρ* = .522; *p* = 0.288 |
| **DRS-2 Memory Raw Score** | *ρ* = -.132; *p* = .557 | *ρ* = -.275; *p* = .303 | *ρ* = .432; *p* = 0.392 |

**Supplemental Table 3: Clusters that demonstrated significantly greater brain activation (*p* < .001) in the control condition compared to the encoding condition (control > encoding) that are 100 voxels or greater in size.** All clusters survived correction for multiple comparisons. Maximum intensity (MI) coordinates are reported in MNI space. X: left-right direction, Y: posterior-anterior direction, Z: inferior-superior direction.

| ***Cluster*** | | ***Maximum Intensity (MI)*** | | | ***Region of Interest*** | | |
| --- | --- | --- | --- | --- | --- | --- | --- |
| ***Number*** | ***Size (Voxels)*** | ***X*** | ***Y*** | ***Z*** | ***Distance from MI (mm)*** | ***Hemi-sphere*** | ***Regions*** |
| 1 | 1257 | 2.1 | -2.4 | 30.8 | 0 | R | Posterior Cingulate Cortex |
|  |  |  |  |  | 2 | L | Posterior Cingulate Cortex |
| 2 | 793 | 30.6 | -90.9 | 2.3 | 0 | R | Lateral Occipital Cortex |
| 3 | 762 | 59.1 | -15.9 | 14.3 | 0 | R | Postcentral Gyrus |
|  |  |  |  |  | 3 | R | Supramarginal Gyrus |
|  |  |  |  |  | 4 | R | Superior Temporal Gyrus |
| 4 | 667 | -60.9 | -35.4 | 27.8 | 0 | L | Supramarginal Gyrus |
| 5 | 520 | -27.9 | -92.4 | -2.2 | 0 | L | Lateral Occipital Cortex |
| 6 | 441 | 38.1 | -36.9 | 54.8 | 0 | R | Postcentral Gyrus |
|  |  |  |  |  | 4 | R | Superior Parietal Cortex |
|  |  |  |  |  | 5 | R | Supramarginal Gyrus |
| 7 | 213 | 24.6 | -38.4 | 59.3 | 5 | R | Superior Parietal Cortex |
|  |  |  |  |  | 5 | R | Postcentral Gyrus |
| 8 | 205 | -20.4 | -14.4 | 65.3 | 1 | L | Precentral Gyrus |
|  |  |  |  |  | 3 | L | Superior Frontal Gyrus |
| 9 | 180 | 32.1 | 8.1 | 2.3 | 0 | R | Putamen |
|  |  |  |  |  | 3 | R | Insula |
|  |  |  |  |  | 7 | R | Pars opercularis |
| 10 | 169 | 57.6 | -11.4 | 6.8 | 0 | R | Postcentral Gyrus |
|  |  |  |  |  | 1 | R | Superior Temporal Gyrus |
|  |  |  |  |  | 3 | R | Transverse Temporal Cortex |
|  |  |  |  |  | 5 | R | Precentral Gyrus |
| 11 | 165 | -56.4 | 11.1 | 23.3 | 0 | L | Precentral Gyrus |
|  |  |  |  |  | 4 | L | Pars opercularis |
| 12 | 149 | -54.9 | -0.9 | 3.8 | 0 | L | Superior Temporal Gyrus |
|  |  |  |  |  | 1 | L | Precentral Gyrus |
|  |  |  |  |  | 4 | L | Postcentral Gyrus |
|  |  |  |  |  | 7 | L | Transverse Temporal |
| 13 | 127 | -42.9 | -2.4 | -2.2 | 0 | L | Insula |
|  |  |  |  |  | 2 | L | Superior Temporal Gyrus |
|  |  |  |  |  | 4 | L | Precentral Gyrus |
|  |  |  |  |  | 7 | L | Transverse Temporal Cortex |
| 14 | 114 | -57.9 | -33.9 | 18.8 | 0 | L | Supramarginal Gyrus |
|  |  |  |  |  | 1 | L | Superior Temporal Gyrus |
| 15 | 105 | -24.9 | -20.4 | 60.8 | 4 | L | Precentral Gyrus |

**Supplemental Table 4: Examining cardiorespiratory fitness as a predictor of ROI beta coefficients, using linear regression analyses controlling for age and sex, across the whole sample of men and women (*N* = 22).** ^†^Denotes *p* < .100. *Denotes significance at the level of *p* < .050. **Denotes significance at the level of *p* < .010. ***Denotes significance at the level of *p* < .001.

| ***Cluster Number*** | ***Hemi-sphere*** | ***Regions*** | ***Estimated V̇O_2max_ – β*** | ***SE*** | ***t-statistic*** | ***p-value*** | ***Delta-R^2^*** |
| --- | --- | --- | --- | --- | --- | --- | --- |
| 1 | R | Isthmus of the Cingulate Cortex | 0.005 | 0.006 | t[18] = 0.875 | *p* = .393, *p_adjust_* for ROIs (7) = .917 | 0.033 |
|  | L | Isthmus of the Cingulate Cortex |  |  |  |  |  |
|  | R | Precuneus |  |  |  |  |  |
| 2 | R | Inferior Parietal Cortex | 0.000 | 0.007 | t[18] = -0.025 | *p* = .980, *p_adjust_* for ROIs (7) = .980 | < 0.001 |
| 3 | L | Inferior Parietal Cortex | 0.006 | 0.005 | t[18] = 1.146 | *p* = .267, *p_adjust_* for ROIs (7) = .917 | 0.065 |
| 4 | L | Lobule VIIa Crus I (Hemisphere) | -0.002 | 0.005 | t[18] = -0.402 | *p* = .692, *p_adjust_* for ROIs (7) = .969 | 0.006 |
|  | L | Lobule VIIa Crus II (Hemisphere) |  |  |  |  |  |
| 5 | R | Lobule VIIa Crus I (Hemisphere) | 0.015 | 0.008 | t[18] = 1.877 | *p* = .077^†^, *p_adjust_* for ROIs (7) = .538 | 0.160 |
|  | R | Lobule VIIa Crus II (Hemisphere) |  |  |  |  |  |
| 6 | L | Lobule VIIa Crus II (Hemisphere) | -0.003 | 0.007 | t[18] = -0.408 | *p* = .688, *p_adjust_* for ROIs (7) = .969 | 0.008 |
|  | L | Lobule VIIa Crus I (Hemisphere) |  |  |  |  |  |
| 7 | R | Fusiform Gyrus | < 0.001 | 0.006 | t[18] = -0.060 | *p* = .952, *p_adjust_* for ROIs (7) = .980 | < 0.001 |
|  | R | Parahippocampal Gyrus |  |  |  |  |  |
|  | R | Lingual Gyrus |  |  |  |  |  |
|  | R | Hippocampus |  |  |  |  |  |

**Supplemental Table 5: Examining cardiorespiratory fitness as a predictor of ROI beta coefficients, using linear regression analyses controlling for age, in women only (*N* = 16).** ^†^Denotes *p* < .100. *Denotes significance at the level of *p* < .050. **Denotes significance at the level of *p* < .010. ***Denotes significance at the level of *p* < .001.

| ***Cluster Number*** | ***Hemi-sphere*** | ***Regions*** | ***Estimated V̇O_2max_ – β*** | ***SE*** | ***t-statistic*** | ***p-value*** | ***Delta-R^2^*** |
| --- | --- | --- | --- | --- | --- | --- | --- |
| 1 | R | Isthmus of the Cingulate Cortex | 0.008 | 0.007 | t[13] = 1.106 | *p* = .289, *p_adjust_* for ROIs (7) = .674 | 0.084 |
|  | L | Isthmus of the Cingulate Cortex |  |  |  |  |  |
|  | R | Precuneus |  |  |  |  |  |
| 2 | R | Inferior Parietal Cortex | 0.004 | 0.007 | t[13] = 0.505 | *p* = .622, *p_adjust_* for ROIs (7) = .871 | 0.019 |
| 3 | L | Inferior Parietal Cortex | 0.009 | 0.006 | t[13] = 1.385 | *p* = .189, *p_adjust_* for ROIs (7) = .662 | 0.125 |
| 4 | L | Lobule VIIa Crus I (Hemisphere) | -0.001 | 0.005 | t[13] = -0.135 | *p* = .895, *p_adjust_* for ROIs (7) = .986 | 0.001 |
|  | L | Lobule VIIa Crus II (Hemisphere) |  |  |  |  |  |
| 5 | R | Lobule VIIa Crus I (Hemisphere) | 0.023 | 0.006 | t[13] = 3.881 | *p* = .002**, *p_adjust_* for ROIs (7) = .013* | 0.532 |
|  | R | Lobule VIIa Crus II (Hemisphere) |  |  |  |  |  |
| 6 | L | Lobule VIIa Crus II (Hemisphere) | 0.004 | 0.006 | t[13] = 0.657 | *p* = .523, *p_adjust_* for ROIs (7) = .871 | 0.028 |
|  | L | Lobule VIIa Crus I (Hemisphere) |  |  |  |  |  |
| 7 | R | Fusiform Gyrus | 0.000 | 0.006 | t[13] = 0.018 | *p* = .986, *p_adjust_* for ROIs (7) = .986 | < 0.001 |
|  | R | Parahippocampal Gyrus |  |  |  |  |  |
|  | R | Lingual Gyrus |  |  |  |  |  |
|  | R | Hippocampus |  |  |  |  |  |
